# Supplementary material for: Recognition of emotion and pain by owners benefits the welfare of donkeys in a challenging working environment
Source: PeerJ. 2023 Aug 8;11:e15747. doi: 10.7717/peerj.15747 (PMC10416770; doi:10.7717/peerj.15747)
Supplement: File S2 [file peerj-11-15747-s002.docx]

1


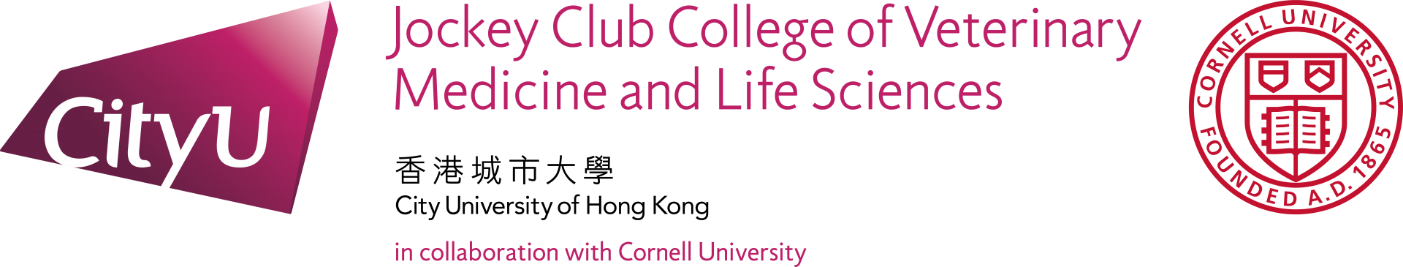


**Demographics**

- **Owner age (years):**

**(a)** less than 31 **(b)** 31-40 **(c)** 41-50 **(d)** More than 50

- **Region:**

**(a)** Swat **(b)** Attock **(c)** Faisalabad **(d)** Cholistan

- **Area:**

**(a)** Rural **(b)** Peri-Urban **(c)** Urban

# Loading Work Data

- **How you assess that the load you are putting on your donkey is practical for them?**

**(a)** By weighing load **(b)** Checking donkey behavior

**(c)** Adding load approximately **(d)** I don’t know

- **Do you use padding under the saddle?**

**(a)** Yes **(b)** No

- **How is the speed of loaded donkey selected?**

**(a)** Chosen by donkey **(b)** triggered by you

- **Does the duration of work per day vary by season?**

**(a)** Yes **(b)** No

- **Is there availability of feed during the working day?**

**(a)** Yes **(b)** No

- **Is there availability of water during the working day?**

**(a)** Yes **(b)** No

# Load associated injuries

- **Have you seen load associated injuries in your donkey?**

1. Wound **(b)** Lameness **(c)** Back pain **(d)** No injuries observed

- **What is the cause of load associated injuries?**

1. Type of load **(b)** Overload **(c)** Practices of loading/unloading

# Owner’s view on loading practices

- **The weight that you put on your animal, is it good for your donkey?**

1. Yes **(b)** No **(c)** I don't know

- **What is the reason that people overload their donkeys?**

1. For more income **(b)** To finish work earlier

- **Have you noticed an increase or decrease in general body condition since you bought this donkey?**

1. No change in body condition **(b)** Body condition increased

**(c)** Body condition decreased

- **Do you think your donkey has emotions?**

**(a)** Yes **(b)** No **(c)** I don't know

- **Do you think your donkey feels pain?**

**(a)** Yes **(b)** No **(c)** I don't know

- **Would you follow loading guidelines (if available) for the benefit of your donkey?**

1. Yes **(b)** No **(c)** I don't know
